# Valve-Sparing Resection of Aortic Papillary Fibroelastoma in an Elderly Patient with Recurrent Stroke

**DOI:** 10.1016/j.cjco.2026.03.003

**Published:** 2026-03-14

**Authors:** Adam Kowalówka, Mikołaj Jodłowski, Anna Bednarek, Radosław Gocoł

**Affiliations:** a Department of Cardiac Surgery, Upper-Silesian Heart Centre, Katowice, Poland; b Department of Cardiac Surgery, Medical University of Silesia, Katowice, Poland; c 1st Division of Cardiology and Structural Heart Diseases, Upper-Silesian Heart Centre, Katowice, Poland

**Keywords:** papillary fibroelastoma, aortic valve, stroke, valve-sparing surgery, elderly

**Papillary fibroelastoma (PFE) is the most common benign cardiac valvular tumour and an important cause of systemic embolization.**^1^ **Left-sided lesions carry the highest embolic risk, with the aortic valve being a frequent location.**^1^^,^^2^ **Although surgical excision is recommended for symptomatic patients, the optimal operative strategy—valve replacement vs valve-sparing resection, remains a topic of debate.**^2^^,^^3^ **We present an elderly patient with recurrent cerebral ischemia caused by aortic valve PFE, successfully treated with valve-sparing excision.**

## Case Presentation

**Figure 1:**
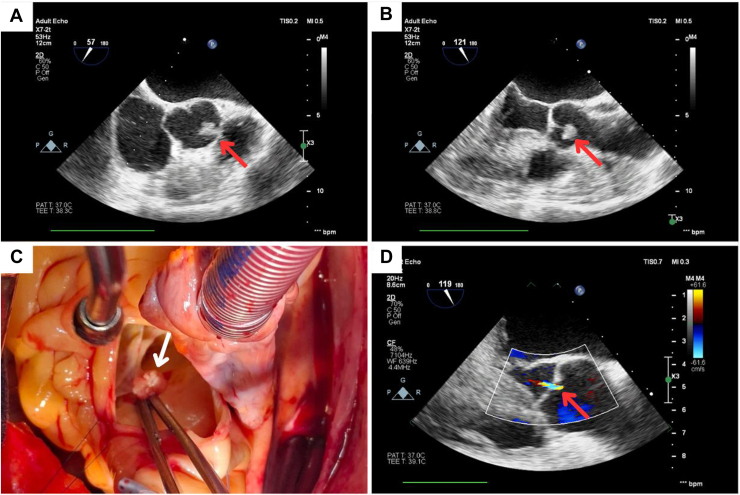
(**A**) Preoperative transesophageal echocardiography (TEE), mid-esophageal aortic valve short-axis view showing the mobile lesion attached to the aortic valve (**red arrow**). (**B**) Preoperative TEE, mid-esophageal aortic valve long-axis view demonstrating the tumour protruding into the left ventricular outflow tract (**red arrow**). (**C**) Intraoperative photograph showing the tumour arising from the left coronary cusp of the aortic valve (**white arrow**). (**D**) Post-excision intraoperative TEE, mid-esophageal aortic valve long-axis view with colour Doppler showing a competent aortic valve without residual regurgitation (**red arrow**).

An 81-year-old man with hypertension, type 2 diabetes, depression, and benign prostatic hyperplasia presented with acute dizziness, horizontal nystagmus, and visual disturbances. The patient had a National Institutes of Health Stroke Scale score of 2. Head computer tomography revealed multiple subacute ischemic lesions in both cerebral hemispheres. Transthoracic echocardiography demonstrated a mobile echodense mass attached to the left coronary cusp of the aortic valve, with preserved left ventricular ejection fraction (55%) and mild aortic regurgitation. Transesophageal echocardiography (TEE) confirmed a highly mobile pedunculated lesion originating from the left coronary cusp, raising suspicion for PFE (Fig. 1A and B; Video 1). Blood cultures remained negative, and coronary angiography showed nonobstructive disease.

Due to recent stroke, advanced age, and anticoagulation requirements related to cardioembolic valve tumour, surgery was initially deferred. However, within 3 hours of discharge, the patient developed recurrent neurologic symptoms. Repeat neuroimaging demonstrated a new cerebellar ischemic lesion. Given recurrent embolization despite medical therapy, urgent cardiac surgery was undertaken.

Surgery was performed via median sternotomy using cardiopulmonary bypass with del Nido cardioplegia. Transverse aortotomy revealed a 3-cm pedunculated, gelatinous, frond-like mass arising from the ventricular surface of the left coronary cusp (Fig. 1C). The remaining leaflets appeared thin, pliable, and noncalcified. The tumour was excised, using an “en bloc” technique, together with a narrow rim of macroscopically normal leaflet tissue to ensure complete removal while preserving structural integrity, rather than by the more commonly employed “shaving” of the leaflet surface. Postresection inspection confirmed sufficient leaflet integrity to maintain valve competence without repair or replacement. Intraoperative TEE showed preserved tricuspid aortic valve geometry without significant residual aortic regurgitation (Fig. 1D; Video 2).

The postoperative course was uneventful. The patient was extubated early, mobilized on postoperative day 1, and discharged on day 6. Transthoracic echocardiography at discharge showed a normally functioning native aortic valve without significant stenosis or regurgitation. Histopathologic examination confirmed PFE, demonstrating avascular papillary fronds with a dense collagenous and elastic core covered by endothelium. No further embolic events occurred during the 2-month follow-up period.

## Discussion

This case highlights several important aspects of PFE management. First, recurrent cerebral ischemia shortly after initial presentation, despite medical therapy, strongly supported a causal link between the tumour and embolization, prompting urgent surgical intervention. This case underscores the need for a low threshold for surgery in patients with PFE and documented embolic events, even elderly patients with comorbidities.^1^^,^^2^

Second, multimodality imaging was crucial. TEE provided detailed characterization of lesion morphology, attachment site, mobility, and valve anatomy, essential information for differentiating PFE from other valvular lesions and assessing feasibility of valve-sparing resection.^4^

Third, this case adds to evidence supporting valve-sparing surgery for aortic valve PFE in appropriately selected patients. Our patient's aortic valve exhibited noncalcified, morphologically preserved leaflets with only mild regurgitation. Complete excision of the tumour with a limited rim of leaflet tissue allowed preservation of native valve geometry and function. This strategy is particularly advantageous in elderly patients, eliminating risks associated with 3 months of anticoagulation after biological valve replavement, or structural valve degeneration requiring reoperation (bioprostheses).^3^^,^^5^

Current guidelines support individualized decision-making based on patient age, comorbidities, valve morphology, hemodynamic performance, and embolic risk rather than tumour size alone.^5^ Despite the relatively large lesion (3 cm), valve-sparing resection remained feasible due to the preserved leaflet structure. This case reinforces an approach in which tumour dimensions alone do not dictate surgical strategy; the stalk of the mass enabled resection without disrupting aortic valve integrity. Instead, decisions regarding valve replacement should be based on comprehensive assessment of leaflet integrity and anticipated durability of native valve competence.

Finally, advanced age should not be considered an absolute contraindication to surgical treatment of PFE when embolic risk is high and expected postoperative quality of life is favourable. With contemporary perioperative management and meticulous surgical technique, valve-sparing excision can be performed safely, as evidenced by the uncomplicated course and the absence of recurrent neurologic events in this patient.

## Conclusion

In patients with aortic valve PFE, surgical treatment should be strongly considered for symptomatic or high-risk lesions, irrespective of chronological age. Decisions regarding valve replacement vs valve-sparing resection must incorporate comprehensive evaluation of leaflet morphology, valve hemodynamic performance, and the overall patient risk profile, rather than tumour size alone. When aortic valve leaflets are noncalcified, structurally preserved, and hemodynamically competent, complete excision with a limited leaflet margin represents a safe and effective alternative to prosthetic valve replacement, preventing further embolic events while preserving native valve function and avoiding long-term prosthetic valve complications.

## Ethics Statement

The study was approved by the Institutional Review Board of the Medical University of Silesia (approval number: PCN/CBN/0052/KB/105/23; approved on June 15, 2023).

## Patient Consent

The authors confirm that patient consent forms were obtained for this article. Written informed consent was obtained from the patient for publication of this case report and accompanying anonymized images.

## Funding Sources

The work was supported by Project European Funds for Silesia 2021-2027, entitled: “Supporting the transformation of the region by strengthening the potential of the Doctoral School of the Medical University of Silesia in Katowice” (NWD/1028/2025).

## Disclosures

The authors have no conflicts of interest to disclose.

**Novel Teaching Points**

• Recurrent cerebral ischemia despite medical therapy in patients with cardiac papillary fibroelastoma warrants urgent surgical intervention regardless of advanced age, as demonstrated by successful valve-sparing resection in this 81-year-old patient with complete resolution of embolic events.
• Tumour size alone should not dictate surgical strategy for aortic valve papillary fibroelastoma; valve-sparing resection remains feasible even for large lesions (3 cm) when leaflets are noncalcified, structurally preserved, and hemodynamically competent, avoiding lifelong anticoagulation or prosthetic valve-related complications.
• Multimodality imaging with TEE is essential for differentiating papillary fibroelastoma from other valvular lesions and determining feasibility of valve-sparing vs valve-replacement surgery by characterizing tumour morphology, attachment site, mobility, and underlying leaflet integrity.
• En bloc tumour excision with a narrow rim of macroscopically normal leaflet tissue, rather than surface shaving, ensures complete removal while preserving native valve function in appropriately selected patients, representing a safe alternative to prosthetic valve replacement in elderly populations.

## Supplementary Material

Video 1 Preoperative transesophageal echocardiography (mid-esophageal short- and long-axis aortic valve views) demonstrating a highly mobile pedunculated lesion attached to the left coronary cusp of the aortic valve.

Video 2 Post-excision intraoperative transesophageal echocardiography (mid-oesophageal long-axis aortic valve view) demonstrating normal leaflet motion and a competent aortic valve without residual regurgitation on colour Doppler following removal of the valvular mass.
